# Predictors of procedural intervention in otolaryngology consultations: a retrospective multivariable analysis of 1,366 cases

**DOI:** 10.1590/1806-9282.20260459

**Published:** 2026-08-24

**Authors:** Serkan Altıparmak, Ayhan Kars

**Affiliations:** 1 Erzincan Binali Yıldırım University, Mengücek Gazi Training and Research Hospital, Department of Otolaryngology – Erzincan, Türkiye.

**Keywords:** Otolaryngology, Referral and consultation, Emergency service, hospital, Surgical procedures, operative

## Abstract

**OBJECTIVE:**

The objective of this study is to evaluate the demographic and clinical characteristics of cases consulted with the ear, nose, and throat department in a tertiary hospital, the consultation indications, and independent factors associated with the need for procedural intervention.

**METHODS:**

This retrospective single-center study included patients undergoing ear, nose, and throat consultation. Demographics, consultation characteristics, indications, imaging, procedures, and hospitalization were recorded. Procedural intervention rates and independent factors associated with intervention requirements were also examined.

**RESULTS:**

Consultations for 1,366 patients were analyzed. The great majority of consultations (76.5%) originated from the emergency department, and procedural interventions were performed in approximately half the cases (48.8%). The most common reasons for consultation were suspected foreign bodies (n=248, 18.2%), nasal fracture (n=231, 16.9%), and epistaxis (n=132, 9.7%). The highest procedural intervention requirements were in the soft tissue trauma group (n=118; 90.7%) and in the epistaxis (87.9%) and nasal fracture (78.3%) cases. Consultation indication was the strongest independent predictor. Epistaxis, trauma-related consultations, and suspected foreign bodies were significantly associated with the need for procedural intervention.

**CONCLUSION:**

Consultation indication was the main determinant of intervention. Epistaxis, trauma-related conditions, and suspected foreign bodies were significantly independently associated with intervention requirements. Early identification of these high-risk indications may assist in the optimization of ear, nose, and throat emergency resource planning and consultation triage.

## INTRODUCTION

Specialty sub-branch consultations are an inseparable component of comprehensive medical care. These contribute significantly to the patient management process by facilitating the sharing of information and expertise among physicians^1^.

Ear, nose, and throat (ENT) consultations constitute an important part of presentations originating from the emergency department, intensive care units, and other clinics in a tertiary center. These consultations include various complaints with very different degrees of severity and urgency. Epistaxis, trauma, foreign bodies, otological difficulties, and airway-related problems represent an important part of these consultations^2^.

Increased demand for health services in recent years and a marked rise in the patient burden in hospitals have also raised the frequency and importance of consultation processes in tertiary centers in particular^3^. Rising patient volumes have made the effective management of specialist consultations requested from the emergency department and other clinics critically important in terms of the quality and sustainability of health services^4^.

A large proportion of ENT consultations in tertiary training and research hospitals involve emergencies and clinical conditions that may require intervention. Revealing the epidemiological characteristics of consultations is important in terms of both clinical resource planning and the development of consultation triage systems^5^. In particular, determining which reasons for consultations are associated with higher intervention rates may contribute to the optimization of shift organization, emergency equipment planning, and patient management algorithms.

The purpose of this study was to evaluate the demographic and clinical characteristics of patients who underwent ENT consultations in a tertiary center, the distribution of consultation indications, and independent factors associated with procedural intervention requirements. Additionally, we intended to employ multivariate logistic regression analysis to determine the effect of intervention requirements on consultation times, the requesting unit, and clinical indications.

## METHODS

Approval for the study was obtained from the Erzincan Binali Yıldırım University Ethics Committee (decision no. 2025-21/02). The research was designed as a retrospective and single-center analysis involving patients consulted with the ENT department of a tertiary training and research hospital between January and December 2025.

The data were retrieved retrospectively from the hospital information management system (HIMS) and electronic patient records. Only official ENT consultation requests made through HIMS were included. Consultations performed by telephone or unofficial verbal consultations were excluded.

Consultations initiated by the emergency department, the internal medicine department, surgical clinics, and intensive care units were included in the study. Patients with incomplete HIMS data, such as detailed history, examination findings, consultation notes, and the procedure performed, were excluded. Following the exclusion of records with deficient data, findings for 1,366 patient consultations were subjected to analysis.

Age, sex, the requesting department, consultation timing (within working hours 08:00–17:00; outside working hours 17:00–08:00), consultation response time, consultation indication, presence of pre-consultation imaging, procedures performed, and hospitalization status were recorded for the cases included in the study. Consultation response time was defined as the time interval between the consultation request recorded in the HIMS and the first ENT evaluation. Reasons for consultation were divided under separate headings: trauma-related conditions (soft tissue trauma, maxillofacial trauma, and nasal trauma), suspected foreign body, epistaxis, tracheotomy cannula evaluation (obstruction, change, bleeding, or discharge), hearing losses, vertigo, otological problems (otorrhagia, otalgia, auricular pathologies, etc.), and infectious/inflammatory conditions. The "other ENT conditions group" was defined as a heterogeneous category including head-neck masses, preoperative evaluation requests, deep neck infections, and other rare otolaryngological pathologies. Consultation indications were categorized based on the primary clinical reason documented in the consultation request and the ENT evaluation notes.

Procedural interventions included nasal cauterization or nasal tampon application for epistaxis, removal of foreign bodies from the nasal cavity, outer ear canal, oropharynx, larynx, or hypopharynx, nasal fracture reduction, suturing resulting from soft tissue traumas, abscess drainage, tracheotomy cannula replacement, and similar invasive procedures. Flexible or rigid endoscopic examinations performed solely for diagnostic purposes were not included in the interventional procedure category. Only procedures requiring active therapeutic intervention were classified as procedural interventions.

If more than one consultation was requested for the same patient, only the first consultation was included in the primary analysis to avoid potential clustering effects.

Continuous variables were presented as median (interquartile range [IQR]; min–max) and categorical variables as number and percentage. The relationship between the consultation time and procedural intervention requirement was assessed using the chi-square test. Multivariate logistic regression analysis was applied to identify independent factors associated with procedural intervention requirements, the results being reported as adjusted odds ratio (OR) and 95%CI values. Variables were selected a priori based on clinical relevance. All statistical analyses were performed on IBM SPSS Statistics version 26.0 software (IBM Corp., Armonk, NY, USA). Since no procedural intervention events were determined in the infectious/inflammatory conditions group, the OR value for this variable could not be reliably computed (complete separation). Statistical significance was set at p<0.05.

## RESULTS

Consultation data for 1,366 patients were subjected to analysis. The median age of the patients for whom consultations were requested was 39 (IQR: 16–64; min–max 0–103), and 61.8% were men. The great majority of consultations originated from the emergency department (76.5%), and more than half (54.2%) were evaluated outside normal working hours. Procedural interventions were performed in almost half the cases (48.8%) (Table 1).

**Table 1:** Demographic and general characteristics of the ear, nose, and throat consultation episodes.

Variable	Value
Total consultation episodes, n	1,366
Age (years), median (IQR; min–max)	39 (16–64; 0–103)
Sex, n (%)	Male: 844 (61.8%); Female: 522 (38.2%)
Requesting department, n (%)	Emergency: 1,045 (76.5%); Internal medicine: 172 (12.6%); Surgical: 89 (6.5%); ICU: 60 (4.4%)
Consultation timing, n (%)	Working hours: 625 (45.8%); Outside working hours: 741 (54.2%)
Imaging before consultation, n (%)	Yes: 440 (32.2%); no: 926 (67.8%)
Procedural intervention, n (%)	Yes: 666 (48.8%); no: 700 (51.2%)
Response time (minutes), median (IQR; min–max)	27 (21–35; 7–197)

ICU: intensive care unit; IQR: interquartile range.

The most common reasons for consultation requests were suspected foreign body (n=248, 18.2%), nasal fracture (n=231, 16.9%), and epistaxis (n=132, 9.7%). The highest procedural intervention rates were observed in patients with soft tissue trauma (107/118, 90.7%), epistaxis (116/132, 87.9%), and nasal fracture (181/231, 78.3%). Trauma-related consultations constituted 30.3% and had high intervention rates (73.4%). Interventional procedures for foreign body removal were performed in 145 (58.5%) of 248 patients. In contrast, the procedural intervention rate in cases of infectious/inflammatory conditions was significantly lower. The "other ENT conditions" category included 162 (11.9%) cases, of which 47 (29.0%) were deep neck infections. Abscess drainage for deep neck infections was the most common procedure among patients requiring procedural intervention in this category (Table 2).

**Table 2:** Distributions of consultation indications and procedural intervention rates.

Consultation indication	n (%)	Procedural intervention, n (%)
Trauma-related consultations (total)	414 (30.3)	304 (73.4)
Soft tissue trauma (lacerations)	118 (8.6)	107 (90.7)
Nasal fracture	231 (16.9)	181 (78.3)
Maxillofacial trauma	65 (4.8)	16 (24.6)
Epistaxis	132 (9.7)	116 (87.9)
Suspected foreign body	248 (18.2)	145 (58.5)
Tracheotomy-related conditions	68 (5.0)	39 (57.3)
*Other ENT conditions	162 (11.9)	33 (20.4)
Otological complaints	91 (6.7)	3 (3.3)
Hearing loss	53 (3.9)	1 (1.9)
Infectious/inflammatory conditions	70 (5.1)	0 (0.0)

* Other ENT conditions included deep neck infections (n=47), head and neck masses, preoperative evaluations, and other uncommon otolaryngological conditions. ENT: ear, nose, and throat.

In terms of the times of consultations, 625 cases were evaluated within normal working hours and 741 outside such hours. The procedural intervention rate was significantly higher in consultations performed outside working hours compared with those performed during working hours (54.5 vs. 41.9%, p<0.001). The median consultation response time was 28 min (IQR: 22–37) during working hours and 27 min (IQR: 21–33) outside working hours. Although the absolute difference was small, response times were significantly shorter outside working hours (p=0.001).

Independent factors associated with procedural intervention requirements were assessed using multivariate logistic regression analysis. Consultation indication emerged as the strongest independent predictor of procedural intervention. Epistaxis (OR 23.26; 95%CI 13.18–41.06; p<0.001), trauma-related consultations (OR 7.46; 95%CI 5.21–10.68; p<0.001), and suspected foreign body (OR 4.21; 95%CI 2.85–6.22; p<0.001) were significantly independently associated with procedural intervention requirements.

The emergency department representing the unit requesting a consultation was also significantly associated with the need for intervention (OR 1.54; 95%CI 1.00–2.36; p=0.048). Otological complaints exhibited a negative correlation with the need for procedural intervention (OR 0.14; 95%CI 0.05–0.39; p<0.001). The variables of age and the time of consultation (within/outside normal working hours) exhibited no independent and significant effect in the multivariate model (Table 3).

**Table 3:** Multivariable logistic regression analysis of factors associated with procedural intervention.

Variable	Adjusted OR	95%CI	p-value
Age (years)	1.00	0.99–1.01	0.893
Sex (male vs. female)	1.34	1.02–1.76	0.035
Consultation timing (outside vs. working hours)	0.93	0.71–1.23	0.629
Requesting department (emergency vs. others)	1.54	1.00–2.36	0.048
Trauma-related consultations	7.46	5.21–10.68	<0.001
Epistaxis	23.26	13.18–41.06	<0.001
Suspected foreign body	4.21	2.85–6.22	<0.001
Otological complaints	0.14	0.05–0.39	<0.001

OR: odds ratio; CI: confidence interval. Reference categories: female sex, consultation during working hours, and non-emergency departments. Infectious/inflammatory conditions were excluded from the multivariable model due to the absence of events (no procedural interventions observed). Dependent variable: Procedural intervention (yes vs. no).

The OR for infectious/inflammatory conditions could not be reliably estimated due to the absence of procedural interventions in that category (complete separation).

Repeated consultations during the study period were also evaluated. More than one ENT consultation was performed for 58 cases (4.2%) among the 1,366 individual patients. The most common causes of presentation among the subjects of repeated consultations were epistaxis and trauma-related conditions. When the 58 patients with repeated consultations were evaluated on an individual basis, a procedural intervention was performed in at least one consultation in 67% of that group. This finding suggests that clinical manifestations with a high intervention requirement also contribute to a high repeat consultation burden.

A total of 63 patients (4.6%) were hospitalized, predominantly for deep neck infections, uncontrolled epistaxis, and vestibular disorders. The procedural intervention rate among the hospitalized patients was 37.1%.

## DISCUSSION

This study provides a large retrospective analysis of ENT consultations in a tertiary hospital and identifies consultation indication as the strongest independent predictor of procedural intervention. Our findings revealed that ENT consultations to a large extent originated from the emergency department, and that approximately half of these cases required procedural interventions. The powerful correlations between epistaxis, trauma-related conditions, and suspected foreign bodies and the need for intervention show that ENT consultations encapsulate acute clinical manifestations requiring procedures. ENT consultation demand has increased in recent years, with substantial growth reported in academic centers, highlighting the importance of understanding consultation patterns for effective workload planning^6,7^.

A previous study reported that ENT consultations commonly originate from the emergency department, with trauma and infections predominating, while epistaxis and rhinological conditions are more frequent in hospitalized patients^8^. Timsit et al. investigated 20,563 consultation patients presenting to the ENT clinic from the emergency department and cited epistaxis, otological problems, and foreign bodies as the most frequent reasons for consultations^9^. Similarly, in the present study, the majority of consultations originated from the emergency department (76.5%), and the most frequent reasons for consultations were suspected foreign body (18.2%), nasal fracture (16.9%), and epistaxis (9.7%). Our findings are largely consistent with the consultation spectrum reported in previous large-scale studies; however, the higher rate of presentations originating from the emergency department and the predominance of trauma cases reveal a difference in our patient profile. This may be attributable to ours being a tertiary center, the regional trauma burden, patient referral policies, and clinical presentation dynamics.

In their retrospective analysis of 518 ENT consultations and resulting intervention rates, Mors et al. concluded that hospital ENT presentations involved a wide clinical spectrum, that the most common reasons were epistaxis, dysphagia, and airway-related conditions (stridor and tracheotomy problems), and that a significant proportion of cases involved clinical manifestations capable of being managed via outpatient evaluation^10^. In addition, the fact that measures such as drug regulation and flexible endoscopic evaluation were included within the scope of intervention in that study makes a direct comparison with the procedural intervention rates in the current research problematic.

Procedural interventions were performed in approximately half of the cases (48.8%) in this study, and consultation indication emerged as the strongest independent predictor of procedural intervention. This may reflect the high proportion of emergency and trauma cases. Epistaxis, trauma-related consultations, and suspected foreign bodies were independently significantly associated with the need for procedural intervention. No procedural intervention was required in patients classified as having infectious or inflammatory conditions. The majority of these cases were managed conservatively with medical treatment and follow-up recommendations, which accounts for the absence of procedural interventions in this group. Jacobs et al. investigated 562 patients with epistaxis and reported that ENT consultations were requested for 48.2% of the patients evaluated in the emergency department, and that nasal packing was applied as an active intervention to 92.4% of the patients who underwent consultations^11^. Another study, examining nasal fractures, reported that 77% of consultations required reduction procedures^12^. A study investigating foreign bodies in the ENT region reported that such bodies were confirmed and removed in 261 (69.4%) out of 376 cases^13^.

These findings suggest that procedural intervention requirements in hospital ENT consultations are largely dependent on the indication. The fact that acute manifestations such as epistaxis, trauma, and foreign bodies are particularly associated with high intervention rates reveals the importance of these clinical conditions within the consultation burden. While consultation requests originating from the emergency department were independently associated with the need for intervention, the relatively low OR and borderline significance level support the idea that clinical content, rather than the requesting unit, was the determining factor. Similarly, although the intervention rate was higher outside normal working hours, the fact that this was no longer significant in the multivariate model suggests that the case type, rather than the time frame, constitutes the determining factor. Although consultation response times were shorter outside working hours, the absolute difference was small and is unlikely to be clinically meaningful. This finding may reflect reduced institutional activity and fewer competing clinical demands during off-hours. It may therefore be concluded that an indication-based approach will be more rational in the organization of consultation processes. In tertiary care hospitals, this approach may be implemented through indication-based triage protocols for ENT consultations. Emergency physicians may prioritize consultations involving epistaxis, trauma, and suspected foreign bodies, which exhibit the highest likelihood of requiring procedural intervention, thereby improving resource allocation and consultation efficiency. Taken together, these findings highlight the central role of consultation indication in determining procedural intervention in hospital ENT consultations.

This study has a number of limitations. First, the single-center design may limit the generalizability of the findings. Consultation patterns and intervention rates may vary depending on institutional protocols, regional trauma burden, and healthcare system characteristics. Therefore, our findings may not be fully generalizable to other healthcare settings. In addition, the data being retrieved from HIMS due to the retrospective study design may have resulted in potential record deficiency or document bias. Furthermore, methodological heterogeneity in the definition of procedural intervention restricts the performance of comparisons between studies.

However, the large sample size and the use of a detailed clinical classification and multivariate logistic regression analysis represent particular methodological strengths.

## CONCLUSION

The need for procedural interventions in patients receiving ENT consultations in a tertiary center is to a large extent dependent on the indication concerned. Acute clinical manifestations such as epistaxis, trauma, and suspicion of a foreign body powerfully and independently predict the need for intervention. These findings show that the early identification of high-risk consultation indications can improve resource planning and consultation triage processes in ENT emergency consultations.

## Data Availability

The datasets generated and/or analyzed during the current study are available from the corresponding author upon reasonable request.
